# Developing global maps of insecticide resistance risk to improve vector control

**DOI:** 10.1186/s12936-017-1733-z

**Published:** 2017-02-21

**Authors:** Michael Coleman, Janet Hemingway, Katherine Ann Gleave, Antoinette Wiebe, Peter W. Gething, Catherine L. Moyes

**Affiliations:** 1 Department of Vector Biology, Liverpool School of Tropical Medicine, Pembroke Place, Liverpool, L3 5QA UK; 2 Malaria Atlas Project, Oxford Big Data Institute, Li Ka Shing Centre for Health Information and Discovery, University of Oxford, Oxford, OX3 7BN UK

**Keywords:** Insecticide resistance, Malaria, Anopheles, Map

## Abstract

**Background:**

Significant reductions in malaria transmission have been achieved over the last 15 years with elimination occurring in a small number of countries, however, increasing drug and insecticide resistance threatens these gains. Insecticide resistance has decreased the observed mortality to the most commonly used insecticide class, the pyrethroids, and the number of alternative classes approved for use in public health is limited. Disease prevention and elimination relies on operational control of *Anopheles* malaria vectors, which requires the deployment of effective insecticides. Resistance is a rapidly evolving phenomena and the resources and human capacity to continuously monitor vast numbers of mosquito populations in numerous locations simultaneously are not available.

**Methods:**

Resistance data are obtained from published articles, by contacting authors and custodians of unpublished data sets. Where possible data is disaggregated to single sites and collection periods to give a fine spatial resolution.

**Results:**

Currently the data set includes data from 1955 to October 2016 from 71 malaria endemic countries and 74 anopheline species. This includes data for all four classes of insecticides and associated resistance mechanisms.

**Conclusions:**

Resistance is a rapidly evolving phenomena and the resources and human capacity to continuously monitor vast numbers of mosquito populations in numerous locations simultaneously are not available. The Malaria Atlas Project-Insecticide Resistance (MAP-IR) venture has been established to develop tools that will use available data to provide best estimates of the spatial distribution of insecticide resistance and help guide control programmes on this serious issue.

**Electronic supplementary material:**

The online version of this article (doi:10.1186/s12936-017-1733-z) contains supplementary material, which is available to authorized users.

## Background

Since the beginning of the century the number of annual deaths attributed to malaria has more than halved due to significant investment in improved case treatment, and insecticide-based vector control [1]. Only through this multifaceted approach will malaria control and elimination succeed. Effective vector control is a key component of this strategy with insecticides playing a central role in most malaria control programmes. The main focus of prevention relies on long-lasting insecticide-treated nets (LLINs) or indoor residual spraying (IRS), with LLINs alone contributing to 68% of all averted cases over the last 15 years [2]. In Africa over 60% of the population at risk are estimated to sleep under a net while 5% are protected by IRS [1]. The efficacy of these interventions may be compromised by both behavioural avoidance and physiological resistance in malaria vectors. Previously the Malaria Atlas Project (MAP) has collated what data is available for vector bionomics, including behaviour [3] for the dominant vectors of human malaria and now MAP aims to address physiological insecticide resistance.

Currently the only insecticides recommended for use on LLINs by the World Health Organization (WHO) are pyrethroids [4], as they have low mammalian toxicity and high insecticidal activity [5]. In 2013 nearly two thirds of IRS programmes world-wide also relied on pyrethroids. This, along with pyrethroid use in agriculture, has resulted in a high selection pressure for pyrethroid resistance [6–8]. The pressure has been sufficiently severe that there is increasing evidence of pyrethroid failure, particularly for IRS. Since 2015 the more expensive organophosphate pirimiphos methyl has largely replaced pyrethroids for IRS.

The history of insecticide resistance detection has been reviewed elsewhere [6, 7], as have the tools and methods used in detecting resistance [9, 10]. Of greater concern are the increased reports on the ineffectiveness of current malaria prevention tools [11–15]. Risk in public health is defined as; ‘the potential for realization of unwanted, adverse consequences to human life health, property or the environment’ [16]. Applying this here, insecticide resistance poses a serious risk to current malaria prevention activities.

In 2012, WHO published the Global Plan for Insecticide Resistance Management (GPIRM) [17] with the aim of raising awareness of insecticide resistance. The goal is that this plan will be supplemented with guidelines, enabling control programmes to develop individually tailored insecticide resistance management strategies. One acute operational difficulty is the lack of nationally representative spatial and temporal comparable data that concurrently measures insecticide resistance and associated mechanisms. This can be attributed to the shortfall of entomologists, lack of appropriate infrastructure and available funding [18].

To date information on the increase in insecticide resistance is rooted in national reporting systems, predominantly driven by the locality of researchers [1, 7]. Previously, two global insecticide resistance databases have been established, IR Mapper collated 4,084 susceptibility data points by 2014 [19] and VectorBase currently provides 5,656 corrected mortality values [20], and WHO has now created a third [21]. These databases all contain differing amounts of resistance data with information, displayed as single points on maps. The online tools provided by each database allow users to visualise information about each data point, such as the species tested or the sample size, but they do not attempt to take account of any of the potential confounding factors within these datasets or the sampling biases that are present. This, combined with under reporting, for example less than half of the malaria endemic countries reported any entomological data last year, highlights the need to take account of potential confounders and biases to produce robust, consistent and comprehensive estimates of resistance that fill the current gaps in the data.

### A new global mapping project

MAP-IR will first collate and assess the available field data on insecticide resistance, then develop a modelling framework to analyse spatiotemporal patterns of resistance. Here the dataset collated so far from published and unpublished sources is described and assessed. The strengths and weaknesses of the available data are discussed and an analytical plan is outlined that mitigates the issues associated with using collated data that was not generated from a single, systematic, global sampling design. The ultimate aim of this work is to provide resistance data that can be combined with information on vector species and disease prevalence to increase our understanding of the impact that resistance has on disease control. Future work based on the data and principles outlined here will generate the tools to help better target interventions and aid with the development of insecticide resistance management plans [21, 22] on a global scale.

## Methods

Resistance data are obtained from three sources; through published articles, by contacting authors, and by contacting the custodians of unpublished datasets. Published articles are identified using the search terms “insecticide resistance” and “anopheles” in the Web of Science database with no date or language restrictions. Currently all articles published up to the end of 2015 that could be obtained have been reviewed and 684 articles containing bioassay results identified. Of the groups contacted, 15 have so far provided unpublished data.

Where possible received data is disaggregated to single sites and collection periods to provide a fine resolution spatial and temporal dataset. Records reporting less than 100% mortality in the susceptible strain were excluded as were records with control mortality above 20% and results from samples that had been through more than one generation in the laboratory. The data fields extracted cover: mosquito collection methods; mosquito identification methods; bioassay conditions including protocol followed, insecticide concentration, exposure period, mosquito generation tested (wild caught, F1 or mixed), and whether a synergist was used; information about the collection site, and information about the data source. Further details on the exact data fields recorded are given in Additional file 1. Sites covering an area less than 25 km^2^ are assigned coordinates in digital degrees using either the coordinates provided with the data, or using contextual information provided about the site to locate it in online gazetteers such as GeoNames and Google Maps. If mosquitoes from multiple sites were pooled for the bioassay, each site is recorded in the database. If an area greater than 25 km^2^ is given and it is not possible to disaggregate this further, the borders of the area are defined using GIS software such as ArcMap or QGIS. In circumstances where the area given is an administrative unit then the borders are taken from the FAO’s Global Administrative Unit Layers [23]. In addition, when resistance mechanism data are provided, such as *kdr* allele frequencies and P450/mixed function oxidase (MFO) test results, this information is linked to the mosquito collection fields and when relevant also to the bioassay fields.

Site coordinates linked to each dataset are checked using GIS software to ensure the coordinates fall on land, in the right country, and that the location of sites matches the description given by the data source. All other fields are checked to ensure each value falls within the expected range and to identify any missing data, which are then requested from the data source.

This data collation is still in process but data has been extracted from all available articles published up to the end of 2015 that met the inclusion criteria. The current dataset has been assessed to inform the next stage of the planned analyses.

In order to visualise apparent trends for the most important class of insecticides, the full dataset was filtered to extract all bioassay records that used a pyrethroid insecticide. The current dataset was examined over three time periods which were chosen based on data availability and the introduction of pyrethroids in agriculture and public health. Each location linked to these bioassays was assigned to the first order administrative division, as defined by the Global Administrative Units Layer for 2013, that the coordinates or polygon fell within. Any locations that spanned more than one administrative unit were excluded. Where the collection date was missing, the date was assumed to be two years before the article publication year, based on the trend seen for records that have a collection date. For the purposes of this exercise, if the number of mosquitoes tested was missing then the number was assumed to be 60, which is the lower quartile value from the full set of records that did report the number tested.

Data from each first order administrative unit for each of the three time periods was then combined to obtain the first and last years that mosquitoes were collected in, the total number of bioassay records (each record represents a unique collection site and period from a unique study), the total number of mosquitoes tested, and the average reported mortality across all of the records. The average mortality was then plotted on a map, and the full data fields are given in Additional file 2.

## Results

### Data availability for standard metrics linked to insecticide resistance

**Fig. 1:**
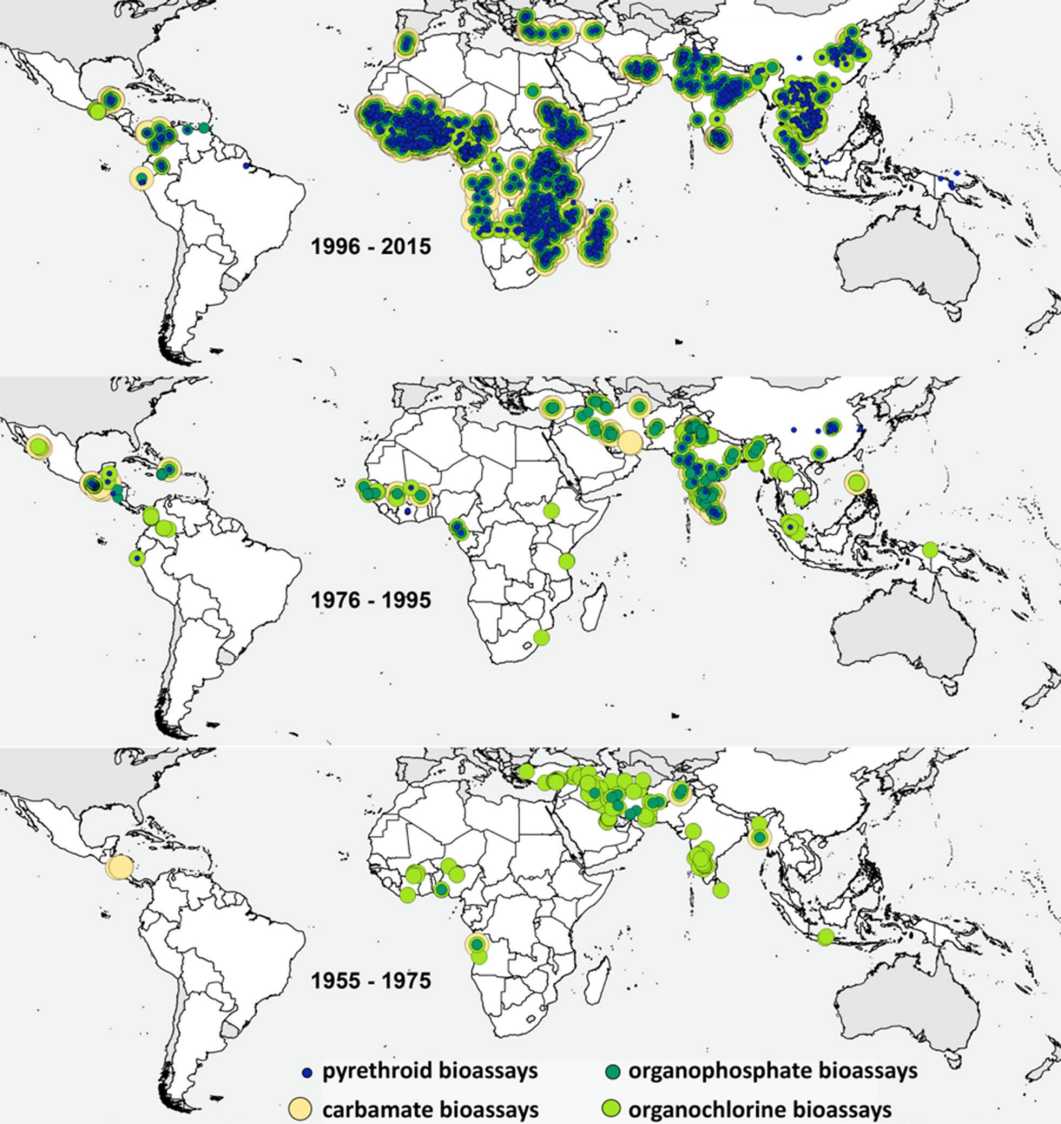
Distribution of the 13,514 insecticide resistance mortality points collected and geopositioned to date

The full current dataset as of October 2016 is summarized in Table 1 and includes insecticide resistance data from 1955 from 71 malaria endemic countries and 74 anopheline species or species complexes. The data includes 1018 survey locations reporting carbamate resistance, 1655 reporting organochlorine resistance, 1056 locations reporting organophosphate resistance and 3127 reporting pyrethroid resistance. These data also cover different insecticides within each class, specifically three carbamates, five organochlorines, eight organophosphates and eight pyrethroids. The methods used to generate these data included CDC bottle assays and ten versions of the WHO bioassay. Figure 1 shows that the data for each of the major insecticide classes are highly clustered, indicating that any analysis of this data needs to account of the clear biases in the location sampled. Temporal bias can also be seen with more data available in more recent years for each class of insecticide.

**Table 1:** The number of records collated to-date

Data type	No. records	No. point locations	No. polygons
Insecticide resistance data from bioassays	14,951	2057	333
kdr allele frequencies	1475	882	25
P450 enzyme activity and gene expression	104	34	1
Esterase enzyme activity	222	123	4

A record is defined as either susceptibility to a specific insecticide or the results of a test for a specific mechanism of resistance, linked to a field-collected species or complex from a defined place and time

### Mapping of pyrethroid resistance over time

**Fig. 2:**
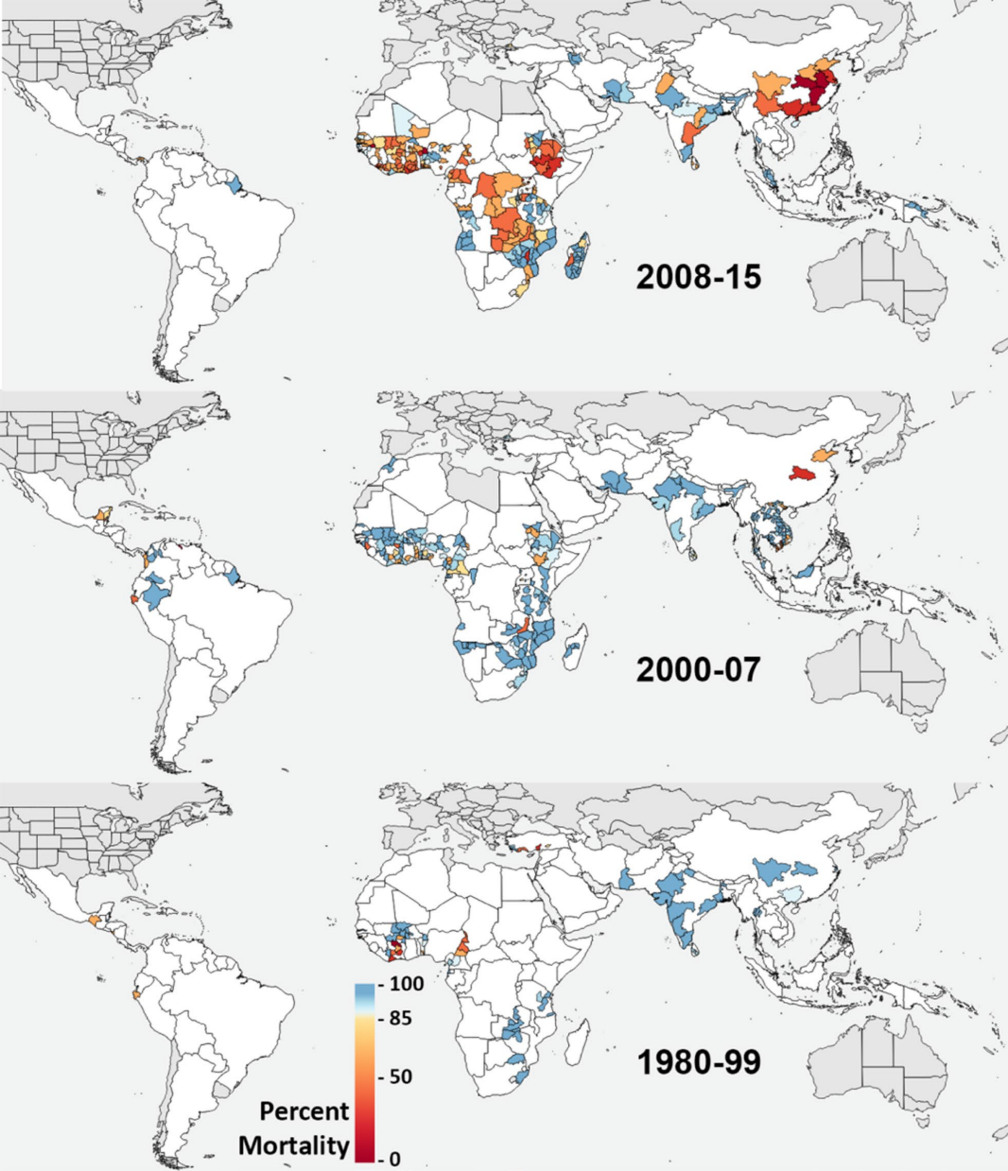
Apparent trends in pyrethroid resistance for the *Anopheles*

The apparent trends of pyrethroid resistance (Fig. 2) were mapped. The base map layers used show malaria endemicity for each time period. Specifically, the 1980–99 map used the 1990 data from the Malaria Elimination Initiative’s time series [24], the 2000–07 map used the WHO’s 2004 data [25] and the 2008–15 map used the 2011 data from the WHO’s 2012 world malaria report [26].

The purpose of the map presented in Fig. 2 was to assess whether there are apparent trends of potential interest that justify a full analysis. The data visualization presented in Fig. 2 should be treated with caution. This map simply displays the raw data without any correction for spatial bias within administrative divisions or temporal bias within each time period. The values shown also combine data from multiple species, insecticides and protocols as noted above. The trend of increased reporting of resistance to pyrethroids over the last 25 years is evident, with areas of Africa that traditionally had no data now reporting.

It is important to note that although the colour scale used in Fig. 2 highlights the thresholds defined by the WHO, the full range of mortality values from 0 to 100% are available for the proposed analyses.

### Data availability for the mechanisms of resistance

**Fig. 3:**
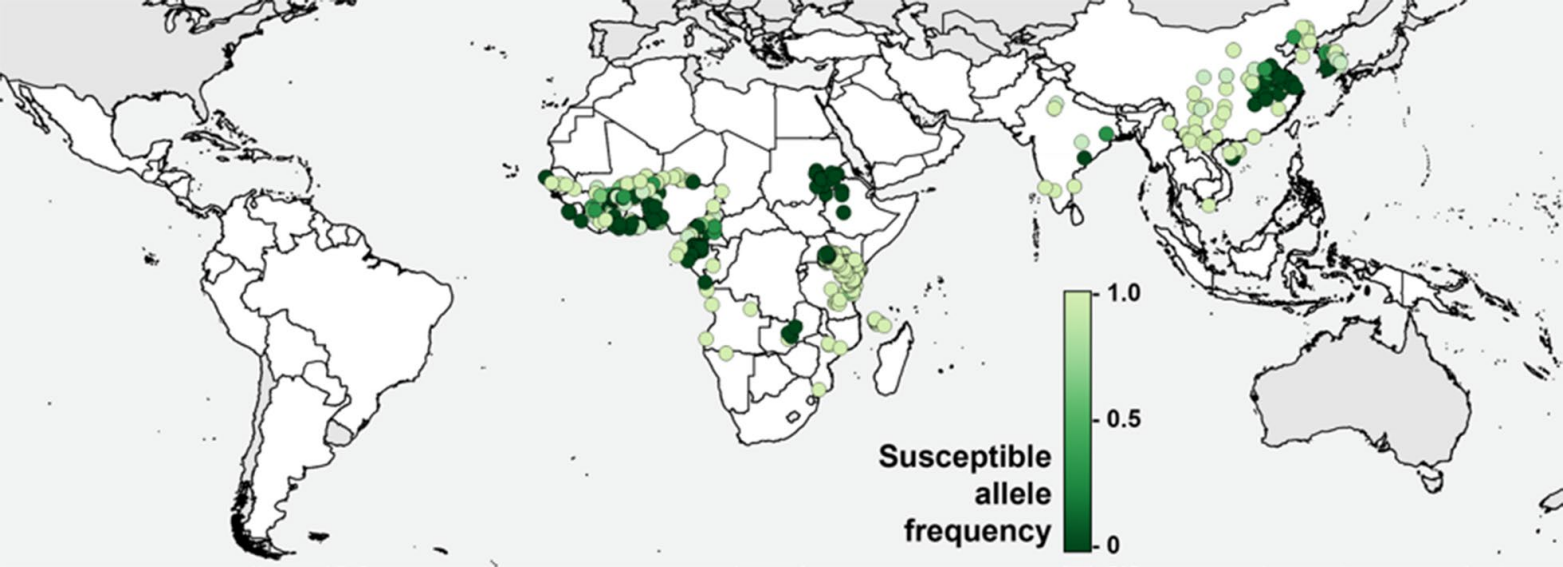
Geographical distribution of *kdr* reports the susceptible allele frequencies

In addition to bioassay data, mechanism data linked to field collections were also extracted. The target site for pyrethroid insecticides is the sodium channel and modification of this, known as *kdr*, can lead to resistance [27]. The full current dataset was filtered to extract all records reporting *kdr* allele data including full genotype frequencies (e.g. the number of homozygotes and heterozygotes), individual allele frequencies and resistant/susceptible allele frequencies. Studies that only provided allele frequencies for a non-representative subset of the population (e.g. bioassay survivors only) were excluded. If data for different species were provided separately, these were combined to give a single value for that site and period. If data for bioassay survivors and dead were provided separately these were combined and weighted by the proportion that had died in the bioassay, to give a single representative value for that site and period. Finally, the susceptible allele frequency was calculated for each record. All frequency values derived from less than 20 mosquitoes tested were excluded. The final dataset current contains 1471 data points at 876 unique locations as shown in Fig. 3.

**Fig. 4:**
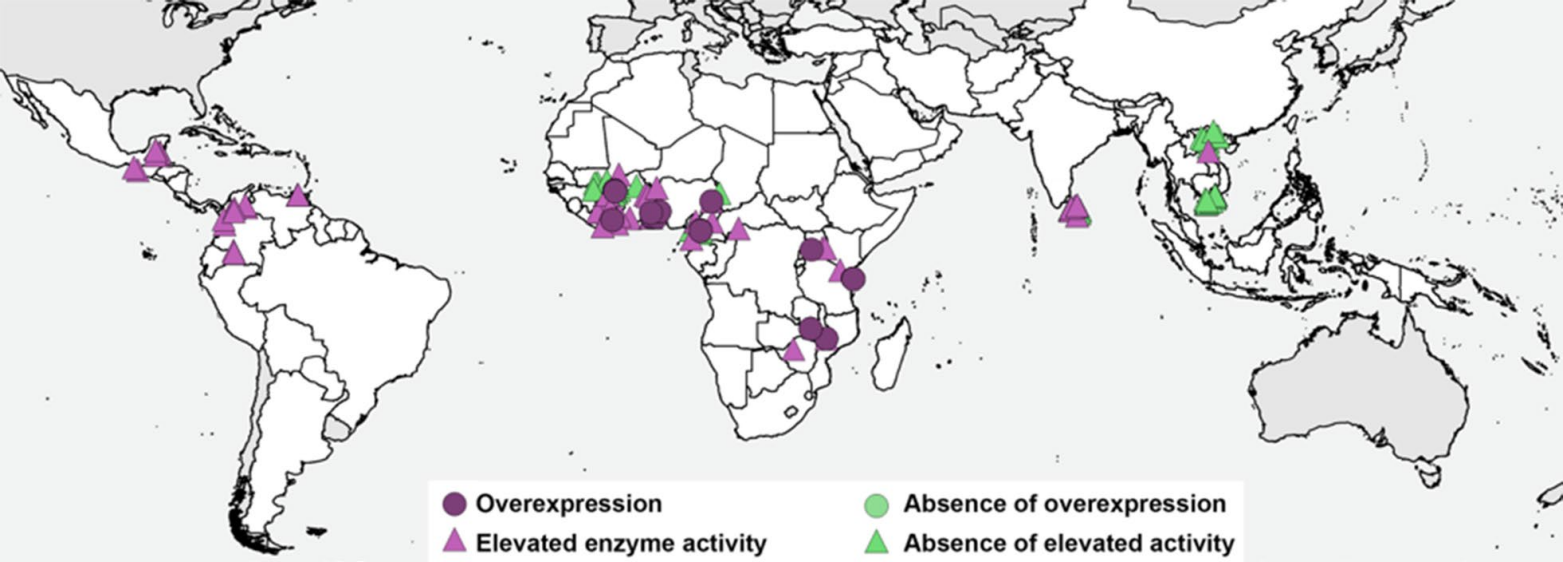
Location of P450/MFO expression reports

Mixed function oxidase is one of the key resistance mechanisms for pyrethroids [27] and has been associated with malaria programme failure [17]. The full current dataset was filtered for all records reporting evidence on cytochrome P450/MFO enzyme activity or gene expression. Each record was classified as either showing significantly higher enzyme activity compared to an appropriate control, not showing significantly higher activity, showing significant overexpression of one or more relevant genes, or not showing overexpression. The current dataset provides 331 P450/MFO data points. The locations of each report of overexpression was then plotted on a map layered on top of reports of high enzyme activity, on top of an absence of overexpression, on top of an absence of high activity. That is, evidence for a ramping up of the P450/MFO enzymes was displayed preferentially over a lack of evidence if both classes of evidence were found at the same location in Fig. 4. Unlike the data for insecticide susceptibility and for *kdr* alleles, it was not possible to derive a single metric for P450/MFO upregulation. The gene expression data covers multiple alleles and the enzyme activity data was recorded using a range of different methods that are difficult to compare.

### Addressing the limitations of the data

The maps presented here allow us to visualize the availability of data and start to see apparent trends, however, an analysis that addresses multiple potential confounding factors (Table 2) is required to elucidate real trends and relationships. It is clear that the only universal metric with the high global data volumes needed to produce comprehensive maps of resistance is phenotypic susceptibility data from standard bioassays. The bioassays methods used include CDC bottle assays [28] and WHO bioassays linked to ten protocol updates [29, 30] meaning any analysis of this dataset needs to incorporate the protocol used as a variable or standardize these data.

**Table 2:** Potential confounders, factors and covariates expected to have the largest effect on observed insecticide susceptibility

Variable	Notes
Sampling bias (spatial)	The dataset was not generated using a single systematic sampling design; the data are highly clustered in geographical space
Sampling bias (temporal)	The dataset did not come from a time series that sampled the same locations at regular intervals; each time period incorporates a different set of sites and much higher data volumes are available for more recent years
Species	The full dataset is linked to 74 malaria vector species and species complexes, however, over half of the bioassay records are linked to members of the *An. gambiae* species complex
Insecticide	Within each insecticide class, different insecticides were tested (6 carbamates, 5 organochlorines, 16 organophosphates, and 8 pyrethroids)
Protocol variation	Corrected mortality values were derived from a mixture of WHO bioassays (using 9 updated protocols) and CDC bottle assays
Exposure dose and duration	The exposure dose and duration used in the bioassays varied although the majority of bioassays used standard doses and times
Generation tested	Population samples were maintained in the laboratory for differing periods, however, only results from bioassays using F0 and F1 generations were included

Data volumes available for *kdr* alleles are much lower and this factor is not strongly linked to the variable of most interest, the efficacy of insecticides. Other mechanisms such as P450/MFO upregulation are more strongly linked to insecticide efficacy, or mosquito mortality, but the volumes of data are currently very low. It may be possible to analyse relationships between mechanism data and the spatiotemporal patterns generated using the bioassay data, especially as mechanism data volumes increase, but these data are insufficient to form the mainstay of the currently planned spatiotemporal analyses.

An initial assessment of the data reveals that spatial variation appears to exist and, as expected, temporal trends are apparent. Sampling intensity is, however, biased in both time and space. To understand these trends it will be important to incorporate both spatial and temporal factors in the analysis to avoid one confounding the other. Insecticide resistance appears to be patchy in space. Spatial patchiness is also seen in malaria prevalence and geostatistical methods incorporating spatial dependence have been shown to provide a robust approach to model these data [31]. These methods have been developed further to incorporate temporal trends and covariates [2], both of which it is expected will to play an important role in insecticide resistance. Specifically, potential drivers of selection such as ITN and IRS use, environmental variables and agricultural use of pesticides will be used as covariates in the model proposed.

The analysis is further complicated by the fact that large numbers of species are represented. Individual anopheline species differ in the likelihood that resistance mechanisms will arise and alleles spread within and between populations so species needs to be included as a factor in the spatiotemporal analyses. The composition of malaria vector species globally forms distinct zones [32] and patterns of resistance may differ among these zones. The planned analysis will therefore consider insecticide resistance within each zone rather than treating this as a single global dataset. Current data volumes are adequate for India, Africa and the Mekong Basin but more data for these areas, particularly historical datasets, will improve the planned analysis and more data for other regions is needed before they can be considered for analysis.

## Discussion

The extent of global insecticide resistance reporting has improved over time (Fig. 1). However, there are still extensive malaria endemic areas for which there are no data yet these data are essential for the selection of appropriate tools for vector control and management of the limited number of insecticides available.

Pyrethroids are a key insecticide class in the fight against malaria as they are still the only class recommended for use on LLINs. The expected impact of a high coverage of LLINs on malaria cases can be lost if efficacy of treated nets on killing resistant mosquitoes is reduced [33]. It has already been noted that the introduction of pyrethroids into South Africa’s IRS control programme had a detrimental effect as pyrethroid resistant *Anopheles funestus* were reintroduced and malaria cases increased [14]. Whereas in the Bioko Island Malaria Control Programme, an initial swap from pyrethroids to carbamates was reversed when it was shown that the *kdr* resistance mechanism alone was not having an operational impact and pyrethroids could still be used to control malaria [34]. This trend is also being observed in LLINs, for example, in Burkina Faso, where local vectors are now 1000 fold resistant to pyrethroids, the personal and community impact of ITNs has been lost [11].

Most programmes rely on a combination of vector control tools. However, countries are now reporting resistance to two or more classes of insecticide with differing resistance mechanisms in different vectors [35, 36]. This makes the development of insecticide resistance management plans challenging and there is a need to potentially target different tools and insecticides to different areas of a country, all of which requires spatial maps of vector species and their insecticide resistance profiles at a granular scale.

Alteration of the pyrethroid target site, *kdr,* is widely distributed but has arisen multiple times in all the vector species tested, with the exception of *An. funestus*, where *kdr* has still to be recorded. The 2000–07 data collected here shows that *kdr* is widespread and corresponds to the period shortly after the scale up of pyrethroid impregnated LLINs, but resistance levels conferred are low. The numbers of reports of *kdr* appear to be declining in recent years, but this is probably because it is less easy to get this information published rather than any evidence that *kdr* testing is declining. This highlights the need for a repository that is able to house both published and unpublished data. GPIRM [17] stresses that metabolic resistance to pyrethroids is probably more important in mosquitoes, however, Fig. 3 shows that this is less well studied. This reflects the difficulty in monitoring metabolic resistance directly in the field, when simple PCR based diagnostics are not available.

### Map discussion

This work has shown that the data volumes of insecticide susceptibility bioassay results are sufficient to allow an analysis of spatiotemporal trends that will yield regional maps and provide modelled predictions for all locations, at a high resolution. The aim while compiling this dataset is to capture the potential confounding factors in addition to the core measures of resistance, linked to location and time data, in order to incorporate these factors into a robust analysis of spatiotemporal trends. The planned Bayesian geostatistical method has been successfully used to model spatiotemporal variation in the prevalence of *Plasmodium falciparum* infections in malaria [2]. Modelling resistance across the vectors that transmit *P. falciparum* and the other human malaria parasites is potentially more complicated and the data requirements for a Bayesian geostatistical model are high. Progress in building a database to feed into this analysis is well under way as presented here but it is noticeable that not all regions are currently well represented and the decision on which regions to include in the model will depend on data availability.

Data sharing is a cornerstone of this work. MAP-IR and VectorBase regularly share non-confidential datasets to maximize the content of both databases. MAP-IR data is also shared with the WHO providing either (i) the data have previously been published, or (ii) the data owners have provided permission for the data to be shared. MAP-IR will utilize the MAP platform [37, 38], allowing users to obtain modelled insecticide resistance risk maps online. MAP-IR differs from previous attempts at mapping insecticide resistance as it is a global initiative that aims to share data from the outset and the largest dataset available is being assembled. In addition to the modelled maps and data, the database of input data (the bioassay and mechanism records described here) will be released into the public domain via the MAP platform. The expected release date for the input data is 1st September 2017, with data being continuously added post-release.

## Conclusions

Insecticide resistance threatens the gains made in malaria control to date. There are currently neither the data nor the resources to generate the information required for control programmes to generate informed decisions regarding vector control policy and insecticide choice. This project will fill some of these gaps which will translate into prolonging the life of old and new insecticides, reduce costs and maintain the gains made in reducing morbidity and mortality in malaria.

## Additional files

**Additional file 1.** Database fields for bioassay records; the data types extracted from each sources are given within a simplified version of the database structure.

**Additional file 2.** Pyrethroid resistance by subnational area for three time periods; the number of bioassay records for each first order administrative division is given for 1980–1999, 2000–2007, and 2008–2015 together with the actual year range for which data are available in each instance, the number of mosquitoes assayed, and the average mortality as shown in Fig. 2.
