# Supplementary material for: Developing global maps of insecticide resistance risk to improve vector control
Source: Malar J. 2017 Feb 21;16:86. doi: 10.1186/s12936-017-1733-z (PMC5320685; doi:10.1186/s12936-017-1733-z)
Supplement: Supplementary file 1 — Additional file 1. Database fields for bioassay records; the data types extracted from each sources are given within a simplified version of the database structure. [file 12936_2017_1733_MOESM1_ESM.docx]

| **Database field** | **Notes** |
| --- | --- |
| Sample ID | A unique identifier for a single collection sample used in a single test (or a set of tests conducted on the same mosquitoes) and linked to up to four citations (see below). |
| ***Field collection table*** | |
| Sample ID | A unique identifier for a single collection sample used in a single test (or a set of tests conducted on the same mosquitoes). |
| Site ID | A unique identifier for each location. |
| Capture method | Four fields are provided to list up to four capture methods if mosquitoes caught using different methods were pooled. |
| Start month | The dates of the field collection for the sample that was tested. |
| Start year | The dates of the field collection for the sample that was tested. |
| End month | The dates of the field collection for the sample that was tested. |
| End year | The dates of the field collection for the sample that was tested. |
| ***Field site table*** | |
| Site ID | A unique identifier for each location. |
| Country |  |
| Site name |  |
| Site type | A ‘point’ location defined as an area <25km2 or a polygon location defined as an area >25km2. If mosquitoes from multiple sites were pooled before they were tested, this is recorded as ‘multi-point’ or ‘multi-polygon’ as applicable. |
| Latitude | Provided for point locations, in decimal degrees. This field is repeated for ‘multi-points’. |
| Longitude | Provided for point locations, in decimal degrees. This field is repeated for ‘multi-points’. |
| GAUL code | An identifier for polygon locations that match a formal administrative division as defined by the UN’s Global Administrative Units Layers. |
| Polygon code | An identifier for polygons that do not match GAUL (see above). |
| ***Species identification table part I: all species*** | |
| Sample ID | A unique identifier for a single collection sample used in a single test (or a set of tests conducted on the same mosquitoes). |
| Species or complex name | Taxonomic classification of the sample that was tested. |
| Identification method | Two fields are provided to list up to two different identification methods. |
| Subset identified | Classifies the sample that was identified as either ‘all’ mosquitoes assayed, ‘survivors’ only, ‘dead’ only, or a ‘mixture’ of survivors and dead but not all of those assayed. |
| Pooled sample | If samples were pooled before mosquitoes were identified, the sample ID for the record linked that has been linked to the identification data is recorded. |
| No. identified | The number of mosquitoes used in the molecular identification tests. |
| Percent identified correctly | The percent of mosquitoes identified as the species given under ‘species name’. |
| ***Species identification table part II:* An. Gambiae *species complex*** | |
| % *An. gambiae/coluzzii* |  |
| % *An. coluzzii* |  |
| % *An. gambiae* |  |
| % *An. arabiensis* |  |
| % *An. melas* |  |
| % *An. merus* |  |
| % *An. quadriannulatus* |  |
| No. g/c identified | If a subset of the sample used in a first identification test that did not split out coluzzii and gambiae was then used in a second test to split out coluzzii and gambiae, the number used in the second test is recorded. |
| % *An. coluzzii / subset* |  |
| % *An. gambiae / subset* |  |
| ***Bioassay table*** | |
| Sample ID | A unique identifier for a single collection sample used in a single test (or a set of tests conducted on the same mosquitoes). |
| Insecticide |  |
| Insecticide class |  |
| Synergist |  |
| Test method | WHO protocol from a specific year, or CDC bottle assay. |
| WHO insecticide concentration (%) |  |
| CDC insecticide concentration |  |
| CDC concentration unit |  |
| Synergist concentration |  |
| Synergist concentration unit |  |
| WHO exposure time (min.) | Duration in minutes. |
| CDC exposure time (min.) | Duration in minutes. |
| Wild caught | ‘adults’ or ‘larvae’ or ‘both’. |
| Generation tested | ‘wild’ = F0, or ‘F1’ or a ‘mixture’ of F0 and F1. |
| Lower age (days) |  |
| Upper age (days) |  |
| Fed status | ‘blood fed’ or ‘non blood fed’. |
| Gravid status |  |
| No. mosquitoes tested |  |
| No. mosquitoes dead |  |
| Corrected % mortality |  |
| ***kdr frequency table*** | |
| Sample ID | A unique identifier for a single collection sample used in a single test (or a set of tests conducted on the same mosquitoes). |
| Subset tested | Defines the overlap with the sample bioassayed as ‘all’, ‘survivors’, ‘dead’, or a ‘mixture’ of survivors and dead but not all of those assayed. |
| Test method | Two fields are provided to list up to two kdr test performed on the same sample. |
| No. tested |  |
| L/L % | Percent homozygous for L allele |
| L/F % | Percent with L and F alleles |
| L/S % | Percent with L and S alleles |
| S/S % | Percent homozygous for S allele |
| L/C % | Percent with L and C alleles |
| C/C % | Percent homozygous for C allele |
| F/S % | Percent with F and S alleles |
| F/C % | Percent with F and C alleles |
| Susc/Susc % | Percent homozygous for susceptible allele |
| Resist/Resist % | Percent with no susceptible allele |
| Susc/Resist % | Percent heterozygous for susceptible allele |
| L1014L % | Frequency of the L allele |
| L1014F % | Frequency of the F allele |
| L1014S % | Frequency of the S allele |
| L1014C % | Frequency of the C allele |
| kdr % | Frequency of resistant alleles |
| ***P450/MFO data table I: enzyme activity*** | |
| Sample ID | A unique identifier for a single collection sample used in a single test (or a set of tests conducted on the same mosquitoes). |
| Subset tested | Defines the overlap with the sample bioassayed as ‘all’, ‘survivors’, ‘dead’, or a ‘mixture’ of survivors and dead but not all of those assayed. |
| Test method | Two fields are provided to list up to two kdr test performed on the same sample. |
| No. tested |  |
| Comparison strain |  |
| Evidence for elevated activity | ‘yes’ or ‘no’ based on significant increase in enzyme activity as defined by the original study. |
| ***P450/MFO data table I: expression*** | |
| Sample ID | A unique identifier for a single collection sample used in a single test (or a set of tests conducted on the same mosquitoes). |
| Subset tested | Defines the overlap with the sample bioassayed as ‘all’, ‘survivors’, ‘dead’, or a ‘mixture’ of survivors and dead but not all of those assayed. |
| Test method | Two fields are provided to list up to two kdr test performed on the same sample. |
| No. tested |  |
| Gene |  |
| Comparison strain |  |
| Fold change |  |
| Evidence for elevated expression | ‘yes’ or ‘no’ based on significantly higher expression as defined by the original study. |
| ***Esterase data table I: enzyme activity*** | |
| Sample ID | A unique identifier for a single collection sample used in a single test (or a set of tests conducted on the same mosquitoes). |
| Subset tested | Defines the overlap with the sample bioassayed as ‘all’, ‘survivors’, ‘dead’, or a ‘mixture’ of survivors and dead but not all of those assayed. |
| Test method | Two fields are provided to list up to two kdr test performed on the same sample. |
| No. tested |  |
| Comparison strain |  |
| Evidence for elevated activity | ‘yes’ or ‘no’ based on significant increase in enzyme activity as defined by the original study. |
| ***Source information table*** | |
| Citation | Four fields are provided to list up to four sources for the data on that sample and the test(s) performed. |
| Citation type | ‘journal article’ or ‘published report’ or ‘unpublished report’ or ‘personal communication’. |
| Release status | ‘published’ or ‘unpublished but permission to release’ or ‘confidential’. |
